# Childhood glaucoma registry in Germany: initial database, clinical care and research (pilot study)

**DOI:** 10.1186/s13104-022-05921-8

**Published:** 2022-02-10

**Authors:** Fidan A. Aghayeva, Alexander K. Schuster, Heidi Diel, Panagiotis Chronopoulos, Felix M. Wagner, Franz Grehn, Nina Pirlich, Susann Schweiger, Norbert Pfeiffer, Esther M. Hoffmann

**Affiliations:** 1 Department of Ophthalmology, University Medical Center of the Johannes Gutenberg, University Mainz, Mainz, Germany; 2 National Centre of Ophthalmology Named After Academician Zarifa Aliyeva, Baku, Azerbaijan; 3 Department of Ophthalmology, University Medical Center Würzburg, Würzburg, Germany; 4 Department of Anaesthesiology, University Medical Center of the Johannes Gutenberg, University Mainz, Langenbeckstraße 1, 55131 Mainz, Germany; 5 Institute of Human Genetics, University Medical Centre of the Johannes Gutenberg, University Mainz, Mainz, Germany

**Keywords:** Childhood glaucoma registry, Congenital glaucoma, Consanguinity, Genetic examination, Questionnaire

## Abstract

**Objective:**

The aim of this prospective pilot study is to establish an initial database to register patients diagnosed with different types of childhood glaucoma and the set-up of a national registry for childhood glaucoma (ReCG) in Germany. 28 children with different types of diagnosed childhood glaucoma, who were admitted and treated at the Childhood Glaucoma Center of the University Medical Center Mainz, Germany were included. Main outcome measures were the type of childhood glaucoma, mean intraocular pressure (IOP) and genetic data of the patients.

**Results:**

The documents and questionnaires for each individual included: informed consent form of the parents, medical history form of the child, patient’s gestational history questionnaire and general anesthesia examination form. Primary congenital and secondary childhood glaucoma were revealed in 11 (39%) and 17 (61%) patients, respectively. The mean IOP measured with Perkins tonometer in all patients under general anesthesia at the time of inclusion was 17.5 ± 11.8 mmHg in the right and 17 ± 8.9 mmHg in the left eyes. In 33% of children with glaucoma mutations in the CYP1B1, FOXC1, LTBP2 and TEK genes were found. The development of specific questionnaires for childhood glaucoma provides detailed baseline data to establish a ReCG in Germany for the first time.

**Supplementary Information:**

The online version contains supplementary material available at 10.1186/s13104-022-05921-8.

## Introduction

Childhood glaucoma is a varied group of rare serious diseases. The incidence of this disorder differs regionally. Approximately one case occurs in 10,000–38,000 live births in Europe, North America, and Australia [1–6]. The highest prevalence can be found in slovakian gypsies (1:1250) [7], in Saudis (1:3030) [1, 8] and in Southern India (1:3300) [9]. Childhood glaucoma is responsible for more than 18% of childhood blindness worldwide [3, 10–14].

Hence, childhood glaucoma is a rare disease in most regions, and etiology and pathogenesis of this disease are only partly known. The standard treatment of congenital glaucoma is the surgical opening of the angle structures to lower intraocular pressure (IOP) [15–17]. The available epidemiological, clinical and therapeutic data on childhood glaucoma are still insufficient [18].

To provide high quality clinical care for patients with childhood glaucoma in Germany, the Pediatric Glaucoma Center was inaugurated at the Department of Ophthalmology of the University Medical Center Mainz, Germany in June 2017. This Centre became soon a competence center taking care of childhood glaucoma patients not only from Germany, but also referred from all over Europe. The aim of this pilot study was to create an initial database of children with glaucoma referred and treated in our Center with maintenance of a national registry for childhood glaucoma (ReCG) in Germany that could be used by other hospitals worldwide. We report demographic, clinical and genetic data of first registered children with diagnosed childhood glaucoma.

## Main text

### Materials and methods

This pilot study included an initial dataset from 29 patients (58 eyes) with different types of childhood glaucoma or childhood glaucoma suspects, diagnosed and treated at the Children Glaucoma Center of the University Medical Center Mainz, Germany from December 2018 to December 2019. The study adhered to the tenets of the Declaration of Helsinki and was approved by the Ethics Committee of the Medical Board of Rhineland-Palatinate, Germany.

A major aim of this pilot study was the development of forms and questionnaires to assess the feasibility and validity before building up a Germany-wide childhood glaucoma registry. These forms and questionnaires were developed and used for acquisition and storage of epidemiological (social data, gestational history, family history) and clinical data of patients diagnosed with childhood glaucoma. The patient information was stored in a database table—electronic form designed as a registry. All the demographic parameters were collected during outpatient examination; clinical parameters were obtained during ophthalmological outpatient examination or examination under general anesthesia.

In our analysis we used congenital glaucoma classification of the World Glaucoma Association [19], that categorizes primary congenital glaucoma (PCG) and the secondary childhood glaucoma (SCG) types, which are associated with other ocular and systemic findings [20, 21].

The health-related quality of life Questionnaires for children at age more than 3 years and adolescents up to 17 years of age (KINDL Questionnaires, Ravens-Sieberer & Bullinger, 2000) were obtained from all included children, depending on their age (from 3 to 6 years; 7–13 years and 14–17 years). The KINDL Questionnaires contained basic questions on age, sex, number of siblings, school education and questions about health-related issues, connected with the glaucoma disease [22].

Indication for surgery was made on a clinical basis under anesthesia, considering the presence of two or more criteria of newly diagnosed children glaucoma or the progression of known disease, according to a novel classification system proposed by the new Childhood Glaucoma Research Network (CGRN) International Childhood Glaucoma Registry. The decision for surgery was immediately discussed with parents in each individual case and informed consent was obtained [21].

To better understand the role of inheritance we performed blood genetic testing of 25 (86.2%) registered children and their parents to identify one or more of the known 24 genes, associated with congenital glaucoma (CNTNAP2, COL11A1, COL4A1, CYP1B1, FOXC1, FOXE3, LMX1B, LOXL1, LTBP2, MAF, MYOC, OPA1, OPA3, OPTN, PAX6, PCMTD1, PITX2, PITX3, PLEKHA7, ST18, TBK1, TEK, TMEM126A, WDR36). For this examination a minimum of 5 ml Ethylenediamine tetraacetic acid (EDTA)-blood from the child under anesthesia (2 ml if newborn or younger than 6 months) and a blood sample (18 ml) from parents was taken [23]. The blood is stored anonymously for up to 10 years according to the German law for biomaterial storage in university medical centers. Besides, the genealogical tree was obtained for every case including special emphasis on consanguinity in the family (a blood relationship between the parents or grandparents).

#### Statistical analysis

Absolute and relative frequencies were computed for dichotomous data, continuous data are presented as mean ± standard deviation. All statistical analysis was conducted with R (R Core Team 2020, R Foundation for Statistical Computing, Vienna, Austria).

### Results

**Fig. 1:**
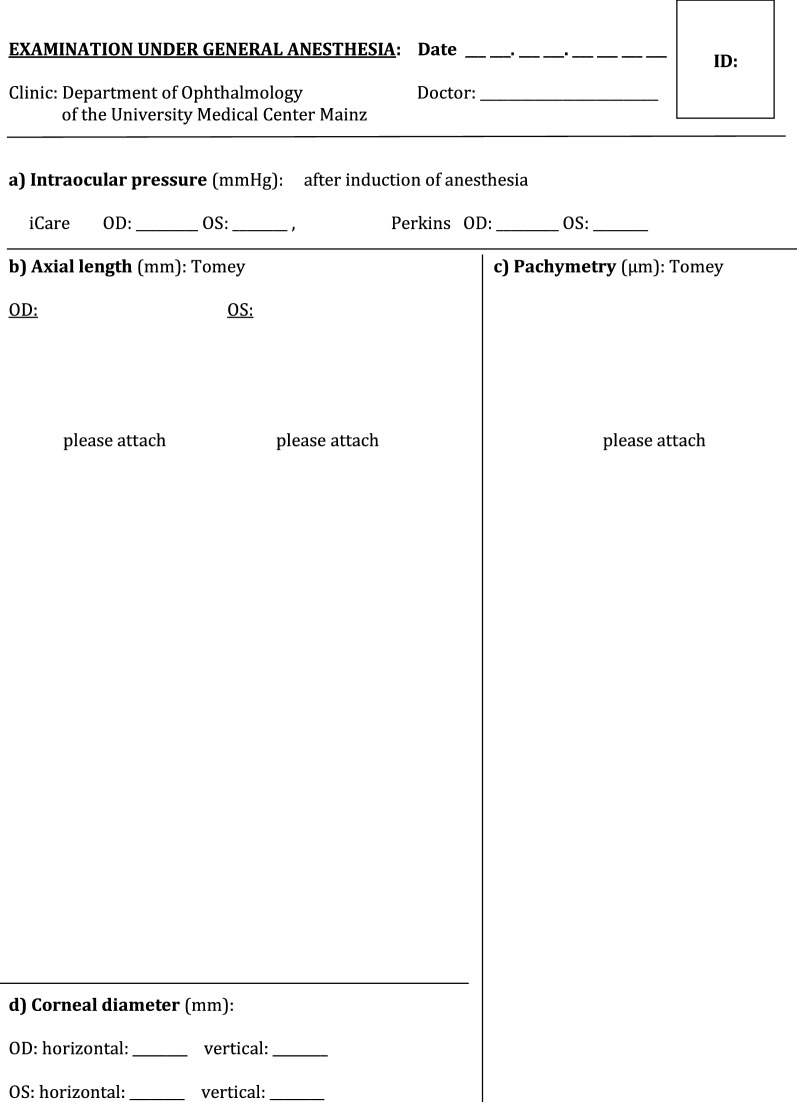

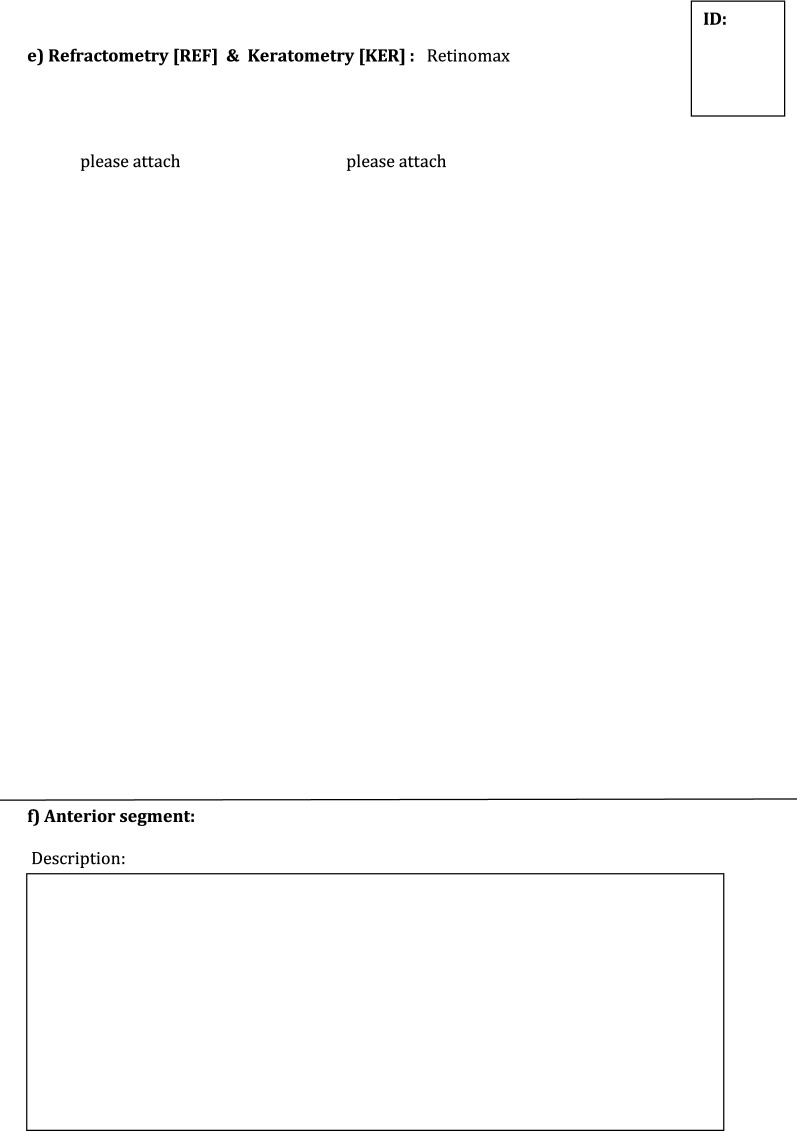

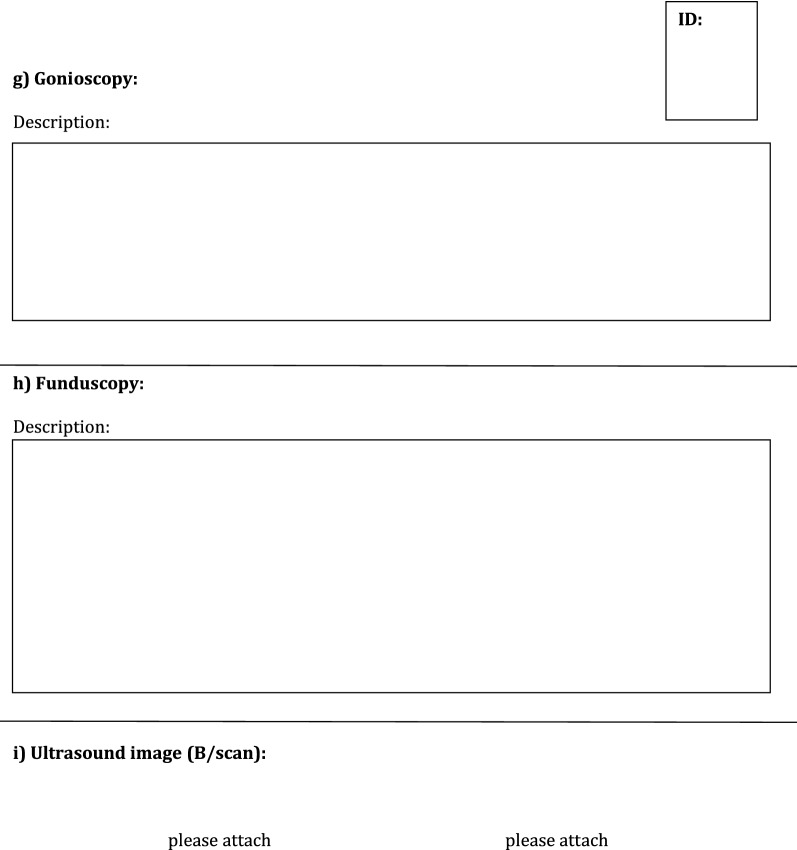
General anesthesia examination form (GAEF)

The created forms and questionnaires included:

1. Agreement form from the parents to participate in the pilot study with purpose of creating ReCG and storing and evaluating the study-related personal health data of their child in a pseudonymized way (i.e., coded without stating name, address, initials etc.,) in paper and electronic form on a secure server of the Mainz University Medical Center as well as a separate agreement form for children younger or older than 12 years. These forms were provided for the parents along with a detailed information letter and the oral explanation of the disease including treatment, registration process (the nature, significance, risks and scope of the register) and other logistic steps, such as possibility to withdraw the participation in the registry at any time, verbally or in writing, without giving any reasons, and without incurring any disadvantages for their child. It was mentioned, that collected data and biomaterial would be deleted after the end of the study, at the latest after 10 years, or at the time of withdrawal from the study.
2. Medical history form/sheet with medical and social history of the child, including patient registration number; birth date; sex; place of birth; place of residence; date and place, where diagnosis of glaucoma was first suspected; date of first examination by eye specialist; affected eye (right, left or both eyes); type of intended therapy (drops or surgery/laser), other diseases and use of medications (see Additional file 1: Fig. S1).
3. Patient’s gestational history questionnaire (general information about pregnancy and details of delivery) including birth dates of both parents, parents’ consanguinity, if present, total number of their biological children, history of childhood or juvenile glaucoma in their families (see Additional file 2: Fig. S2). This survey aimed at possible connections between risk factors in pregnancy and the incidence of childhood glaucoma. It could be completed within about 15 min.
4. Form documenting the examination of the child under general anesthesia (GAEF), containing separate fields for: IOP measured with a hand-held applanation tonometer (Perkins, Haag-Streit, UK) and (Icare, Icare, USA); axial length measured by A-scan ultrasonography; central cornea thickness (AL-3000 Biometer/Pachymeter, Tomey Co, Nagoya, Japan and iPac™ Pachymeter Reichert Inc., USA); corneal diameter—horizontal and vertical; refraction and keratometry parameters (radius of corneal curvature) (Retinomax K-plus2, Nikon Inc., Japan); examination of anterior segment of the eye under the operating microscope (corneal haze, opacification, scarring, Haab’s striae, iris, anterior chamber, and lens abnormalities); gonioscopy; fundus examination with pupil dilation and assessment of optic nerve disc and cup-to-disc ratio (indirect ophthalmoscope, 20 Diopter lens); ultrasound B/scan image (Fig. 1).

In order to reduce interference of depth of anesthesia with IOP values [24, 25], IOP was always measured within the first minutes after initiation of anesthesia. Time point of IOP measurements, method of anesthesia, detailed description of anterior segment and gonioscopy findings, surgical status of the eye and signs of secondary glaucoma were thoroughly documented in the extended GAEF (see Additional file 3: Fig. S3). Examination under anesthesia was performed in 23 (79%) patients. Fundus examination was difficult to perform in 29 (50%) eyes, because of poor view to the posterior segment due to opacity of ocular media. In one patient with large cornea the glaucoma diagnosis was rejected during examination under anesthesia and a CHRDL1 gen, associated with megalocornea, was later identified during genetic blood examination. Demographic and clinical data of all patients with diagnosed childhood glaucoma are shown in Tables 1 and 2. Childhood glaucoma diagnosis was confirmed by considering all ocular, associated ocular, and systemic anomalies. PCG and SCG were revealed in 11 (39%) and 17 (61%) childhood patients, respectively. Bilateral glaucoma was diagnosed in 9 (82%) and 10 (59%) patients with PCG and SCG, respectively. The most common cause of SCG was Peters anomaly (41%) (see Additional file 4: Table S1).

**Table 1:** Descriptive statistics of demographic and genetic data of all patients with diagnosed childhood glaucoma

Number of patients, PCG/SCG	28; 11/17
Age at presentation	
Mean ± SD (range), months	33.8 ± 36.5 (1–125)
Mean ± SD (range), years	2.8 ± 3 (0.07–10.41)
Age at time of suspected glaucoma diagnosis, months	
Mean ± SD (range)	12.3 ± 24.9 (0–119.3)
Sex, n (%)	
Female/Male	18 (64%)/10 (36%)
Country of origin, mother/father, n (%)	
German	14 (50%)/14 (50%)
East European countries	5 (18%)/6 (21%)
Asia	7 (25%)/7 (25%)
Africa	2 (7%)/1 (4%)
Country of birth, n (%)	
German	21 (75%)
East European countries	4 (14%)
Asia	3 (11%)
Laterality, n (%)	
Unilateral/Bilateral	9 (32%)/19 (68%)
Unilateral/Bilateral, PCG	2 (18%)/9 (82%)
Unilateral/Bilateral, SCG	7 (41%)/10 (59%)
Mean number of performed eye surgeries	2 ± 3.7
Mean number of eye surgeries, performed at glaucoma department Mainz	2 ± 3.2
Positive family history, n (%)	6 (21%)
Consanguinity, n (%)	4 (14%)
Risk factors during pregnancy, n (%)	
Smoking	2 (7%)
Alcohol drinking	3 (11%)
Genetic examination	24 (86%)
Revealed genes’ mutations with their type and location, n (%)	12 (50%) out of 24
CYP1B1 (2 compound heterozygous; 3 homozygous; 2p22.2)	5 (21%)
SOX11 (heterozygous; 2p25.2)	1 (4%)
TEK (heterozygous; 9p21.2)	1 (4%)
CRYBB3 (heterozygous; 22q11.23)	1 (4%)
FYCO1 (homozygous; 3p21.31)	1 (4%)
FOXC1 (heterozygous; 6p25.3)	1 (4%)
GJA8 (heterozygous; 1q21.2)	1 (4%)
LTBP2 (compound heterozygous; 14q24.3)	1 (4%)

SD, standard deviation; PCG, primary congenital glaucoma; SCG, secondary childhood glaucoma

**Table 2:** Descriptive statistics of clinical data of patients (n = 28) with diagnosed childhood glaucoma

Data/eye	Right, n = 23	Left, n = 24
Type of childhood glaucoma		
PCG	9	11
SCG	14	13
Presence of IOP-lowering therapy	19 (83%)	21 (88%)
Topical monotherapy	6 (26%)	5 (21%)
Topical combined therapy	12 (52%)	15 (62%)
2 groups of hypotensive drops	9 (39%)	8 (33%)
≥ 3 groups of hypotensive drops	3 (13%)	7 (29%)
Acetacolamide—in one patient	1 (4%)	1 (4%)
Surgical status of eye		
Untreated	11 (48%)	9 (38%)
Previous TO	3 (13%)	3 (13%)
Previous TE	1 (4%)	2 (8%)
Previous Coco or Cyclocryocoagulation	–	1 (4%)
Previous multiple glaucoma surgeries	7 (30%)	8 (33%)
Previous drainage device (Ahmed valve)	1 (4%)	1 (4%)
Mean IOP (SD, range), mmHg		
Perkins	17.5 ± 11.8 (5–50)	17 ± 8.9 (5–43)
iCare	23.3 ± 18 (7–68.5)	20.6 ± 9.4 (8.2–38.7)
Mean axial length (SD), mm	22.3 ± 3.5	23.5 ± 3.8
Mean corneal diameter (SD), mm		
Horizontal	12.4 ± 2.7	11.9 ± 2.5
Vertical	11.3 ± 3.1	10.9 ± 3.2
Mean CCT (SD), µm	623 ± 110.3	632 ± 123.7
Refraction, diopters		
Mean sphere (SD)	1.43 ± 9.8	− 0.23 ± 8.6
Mean cylinder (SD)	− 2.42 ± 2.2	− 3.73 ± 2.6
Keratometry, mm		
Mean K1 (SD)	7.8 ± 0.8	8.4 ± 0.8
Mean K2 (SD)	7.3 ± 0.7	7.9 ± 0.8

PCG, primary congenital glaucoma; SCG, secondary childhood glaucoma; TO, trabeculotomy; TE, trabeculectomy; Coco, cyclophotocoagulation; SD, standard deviation; IOP, intraocular pressure; CCT, central corneal thickness

In 8 (33%) children with childhood glaucoma one of the following congenital glaucoma associated genes (CYP1B1, FOXC1, LTBP2 and TEK) was confirmed by the genetic examination. The most frequently identified mutated gene was CYP1B1 located on chromosome 2p22-p21 (locus GLC3A) (Table 1, see Additional file 4: Table S1). In total, 24 (86%) patients received IOP-lowering therapy and 17 (61%) children underwent glaucoma surgery (Table 2). In 8 (29%) childhood patients were performed multiple glaucoma surgeries.

One patient with SCG associated with Peters anomaly underwent combined XEN-Baerveldt drainage device implantation followed by revisions and lensectomy with synechiolysis after multiple glaucoma surgeries, including Ahmed valve implantation. We performed anterior chamber washout in one patient with hyphema after catheter-assisted 360° trabeculotomy.

Due to inability of IOP control, in total, 4 (24%) out of 17 patients underwent revisions or repeat glaucoma surgeries within 6 months after initial surgery in our Centre.

### Discussion

Our pilot investigation describes the features of the first registry for childhood glaucoma in Germany. We developed special forms and questionnaires for data acquisition that can be used to ascertain the demographic and clinical characteristics of different types of childhood glaucoma.

There are different registries in almost all other areas of ophthalmology worldwide [26–31], including several glaucoma registries [32–35], but only few congenital glaucoma registries [1, 3, 21].

Lopes et al. designed an online form at the Department of Ophthalmology and Visual Sciences of the Federal University of São Paulo to create a registry of patients with congenital glaucoma. The pilot study included 72 children with childhood glaucoma, 61.5% with PCG, and 38.5% with secondary congenital glaucoma, respectively. Most patients with secondary congenital glaucoma were secondary to congenital cataract (61%) [3]. In contrast, most of our SCG patients had Peters anomaly.

Thau et al. presented a review of a new classification system for childhood glaucoma, its use to date, and its application for international research. This classification has become the first International Consensus Classification in 2013 and is used by the new Robison D. Harley, MD CGRN International Pediatric Glaucoma Registry. This registry attempts to overcome the inherent challenges of studies on rare disorders due to limited study size and acts as a centralized database for clinical data provided by sites across the globe to allow large-scale studies on childhood glaucoma [21].

Alanazi et al. determined the demographic and clinical distribution of primary and secondary congenital glaucoma in 180 patients with congenital glaucoma in the Middle East by using data from a registry at King Khaled Eye Specialist Hospital, collected between 2001 and 2003 (29 months). This registry excluded cases with acquired childhood glaucoma. According to their results, 30% of all patients had a positive family history and consanguinity was recognized among 58.9% patients [1].

Different from Arabic and Pakistani populations in which consanguineous marriages are the major contributing factors to the high incidence of recessively inherited PCG [1, 5, 14, 36], we found a much lower frequency of consanguinity in our study (21% of positive family history and 14% of presence of consanguineous marriages). These percentages are comparable to those, reported in the U.S. and Australia [2, 4]. In one third of included childhood glaucoma patients genetic testing identified one of the genes that are associated with congenital glaucoma, confirming the importance of genotyping this disease [7, 36].

Bilateral disease was present in our study in 82% patients with PCG and was higher than the incidence of bilateral PCG (58%), reported by Bayoumi [37]. We observed unilateral disease more frequent in patients with SCG than in PCG, as previously reported [1, 20, 38].

### Conclusions

In summary, this pilot study at the Children Glaucoma Center of the University Medical Center Mainz provided for the first time in Germany detailed baseline data and is aimed at a national registry on different types of childhood glaucoma.

The planned registry will allow to perform various types of registry-based analyses as well as a better understanding of the clinical course of the disease and identification of risk factors. Registered data can provide valuable information on epidemiology, clinical phenotypes and genetics in childhood glaucoma blindness in Germany.

## Limitations

The limitation of this registry was the collection of medical history only by an interview with the child’s parents and not by review of their medical documentation which was not available in most cases. Hence, no complete verification of the family and gestational history is possible in the questionnaires. Due to the lack of a documented description of previous surgical techniques, in most cases it was also impossible to confirm the use of certain surgical agents and conduct a detailed descriptive analysis of interventions performed. We did not investigate differences in age, sex, and ethnical distribution in our study cohort, since it is a pilot study with a limited number of patients in both the PCG and SCG group.

## Supplementary Information

**Additional file 1: Figure S1.** Medical history form with medical and social history of the child.

**Additional file 2: Figure S2.** Patient’s gestational history questionnaire (general information about pregnancy and details of delivery).

**Additional file 3: Figure S3.** Extended general anesthesia examination form (extended GAEF). PHPV = Persistent hyperplastic primary vitreous; ROP = Retinopathy of prematurity; FEVR = Familial exudative vitreoretinopathy.

**Additional file 4: Table S1.** Causes and genetic data of secondary childhood glaucoma in Germany.

## Data Availability

The authors confirm that the data supporting the findings of this study are available within the article and its Additional files. This manuscript contains part of the doctoral thesis of Dr. Heidi Diel named “Results of a pilot study at the Department of Ophthalmology of the University Medical Center Mainz to establish a nationwide registry for childhood glaucoma in Germany” at the University Medical Center of the Johannes Gutenberg-University Mainz. The registry datasets generated and analyzed during the current study are available from the corresponding author on reasonable request.

## Abbreviations

ReCG: National registry for childhood glaucoma
IOP: Intraocular pressure
GAEF: General anesthesia examination form
PCG: Primary congenital glaucoma
SCG: Secondary childhood glaucoma
CGRN: Childhood Glaucoma Research Network
EDTA: Ethylenediamine tetraacetic acid
