# Supplementary material for: Childhood glaucoma registry in Germany: initial database, clinical care and research (pilot study)
Source: BMC Res Notes. 2022 Feb 10;15:32. doi: 10.1186/s13104-022-05921-8 (PMC8830121; doi:10.1186/s13104-022-05921-8)
Supplement: Supplementary file 1 — Additional file 1: Figure S1. Medical history form with medical and social history of the child. [file 13104_2022_5921_MOESM1_ESM.pdf]

14.06.2017

Anamnese: Date: ___ ___ . ___ ___ . ___ ___ ___ ___ (dd/mm/yyyy) **Qo-ID:** ___________

Clinic: ______________________________ Doctor: ____

Date of birth: ___ ___ . ___ ___ . ___ ___ ___ ___ (day / month / year)

Sex: O male O female

Country of origin: O Germany O Other: ________________________ (Country)

Postcode of the residence place in Germany: ___ ___ ___ ___ ___

Date of suspected glaucoma diagnosis: ___ ___ . ___ ___ . ___ ___ ___ ___ (day/month/year)

By O Eye specialist O Pediatrician O Clinic: ____________________________

Date of first exam by eye specialist: ___ ___ . ___ ___ . ___ ___ ___ ___ (day/ month/year)

Eye diagnosed with glaucoma (Parents information): O right eye O left eye

Therapy since first diagnosis: O Eye drops O No O Surgery (inclusive Laser)

In case of eye drops: Active substance and frequency: ______________________________

Eye received therapy: O right eye O left eye

In case of surgery: where, when and which surgeries were performed:

____

____

____

____

Other diseases of child (not only in the eye): O no O yes

If yes, which: ____

____

Taking medications (except eye drops): O no O yes

If yes, which: ____
