# Supplementary material for: Childhood glaucoma registry in Germany: initial database, clinical care and research (pilot study)
Source: BMC Res Notes. 2022 Feb 10;15:32. doi: 10.1186/s13104-022-05921-8 (PMC8830121; doi:10.1186/s13104-022-05921-8)
Supplement: Supplementary file 2 — Additional file 2: Figure S2. Patient’s gestational history questionnaire (general information about pregnancy and details of delivery). [file 13104_2022_5921_MOESM2_ESM.pdf]

Who answers the questionnaire? O Mother O Father Qo-ID: _______

Birth date of mother: ___ ___ ___ ___ (year), Birth date of father: ___ ___ ___ ___ (year)

Are there any relations between you? O Yes O No

If yes, are you: O Cousin/Cousin O _____________

Do you have other biological children? O Yes O No

If yes, how many children? __________ boys __________ girls

Did someone in your family (you, your partner, your parents, your other children, someone else) have glaucoma in childhood (< 18 years old)? O Yes O No

Have someone been blind in a young age (< 18 years old)? O Yes O No

To delivery:

Was your child O premature O normal born, or O post-term baby?

In which week of pregnancy was your child born? ____________

Birth weight of your child: ___________ (gram), Birth size of your child: ___________ (sm)

To pregnancy:

Did the pregnancy occur

O naturally or O through artificial insemination?

Did the mother of your child smoke during the pregnancy? O Yes O No

If yes, how many cigarettes did she smoke on average per day?

______ cigarettes/day

Did the mother of your child drink alcohol during the pregnancy? O Yes O No

If yes, how much alcohol did she drink on average per week?

_________ bottles of beer/week, _________ bottles of wine/week

O Enjoyment of higher percentage alcoholic beverages

Did the mother of your child use drugs during the pregnancy? O Yes O No

Did the mother of your child use medications during the pregnancy?

O Yes O No

If yes, which medications and in which week of pregnancy?

____

Did the mother of your child take folic acid prophylaxis therapy before or during the pregnancy?

O Yes O No
