# Supplementary material for: Childhood glaucoma registry in Germany: initial database, clinical care and research (pilot study)
Source: BMC Res Notes. 2022 Feb 10;15:32. doi: 10.1186/s13104-022-05921-8 (PMC8830121; doi:10.1186/s13104-022-05921-8)
Supplement: Supplementary file 3 — Additional file 3: Figure S3. Extended general anesthesia examination form (extended GAEF). PHPV = Persistent hyperplastic primary vitreous; ROP = Retinopathy of prematurity; FEVR = Familial exudative vitreoretinopathy. [file 13104_2022_5921_MOESM3_ESM.pdf]

Examination under general anesthesia: Date __ __. __ __. __ __ __ __ Qo-ID: _____

Clinic: ____

Intraocular pressure in mmHg: OD: ________ OS: ________ Time point: _____ min after induction of anesthesia

Method: O Perkins O Schiotz O other __________

Method of anesthesia: O Endotracheal intubation: O Laryngeal mask O Gas: ________;
O Propofol O other: ______

Refractometry: O Retinomax O Skyascopy O other device: ________

OD: _____ Dpt. Sphere / _____ Dpt. Cylinder / ________° Axis

OS: _____ Dpt. Sphere / _____ Dpt. Cylinder / ________° Axis

Keratometry: O Retinomax O other device: ________

OD: _____ mm / _____ mm / ________° Axis

OS: _____ mm / _____ mm / ________° Axis

Corneal diameter:

Vertical OD: _____ mm OS: ______ mm Horizontal OD: _____ mm OS: ______ mm

Pachymetry: Mean out of O 1-3 O 4-6 O 7-9 O ≥10 O Device: __________

OD: _______ µm OS: ________ µm

Axial length: O Tomey-________ O other device: ________

OD: __________ mm OS: __________ mm

Anterior segment:

	**OD:**	**OS:**
Megalocornea	O Yes O No	O Yes O No
Haab's striae	O Yes O No	O Yes O No
Corneal stromal opacification	O Yes O No	O Yes O No
Endothelial opacification	O Yes O No	O Yes O No
Posterior embryotoxon	O Yes O No	O Yes O No
Ectropion uveae	O Yes O No	O Yes O No
Iris defects	O Yes O No	O Yes O No
Cataract	O Yes O No	O Yes O No
Other anomaly	O Yes O No	O Yes O No

Signature of doctor (and readable name):

Please turn over!

Funduscopy: Examination feasible: O OD O OS

Photo of optic nerve head O OD O OS obtained

	OD:	OS:
Glaucomatous optic nerve head	O Yes O No	O Yes O No
Abnormal macular reflex	O Yes O No	O Yes O No
Other pathology	O Yes O No	O Yes O No

OD:
CDR: ___________ horizontal

CDR: ___________ vertical

OS:
CDR: _______________ horizontal

CDR: _______________ vertical

Gonioscopy:

	OD:	OS:
Anterior chamber angle can be assessed circularly	O Yes O No	O Yes O No
Anterior chamber angle dysgenesis	O Yes O No	O Yes O No
Peripheral anterior synechiae (gonio)	O Yes O No	O Yes O No
Other anomaly	O Yes O No	O Yes O No

Which:

Known congenital glaucoma/previous surgeries: O OD O OS

Diagnosis:

OD:

O Primary congenital glaucoma

O Secondary glaucoma due to:
- O Aphakia
- O Uveitis
- O Ocular trauma
- O Aniridia
- O Axenfeld-Rieger anomaly
- O Peters Anomaly
- O Sclerocornea
- O Sturge Weber Syndrome
- O Neurofibromatosis type 1
- O Lowe Syndrome
- O Posterior anomalies (PHPV, ROP, FEVR)
- O other:

OS:

O Primary congenital glaucoma

O Secondary glaucoma due to:
- O Aphakia
- O Uveitis
- O Ocular trauma
- O Aniridia
- O Axenfeld-Rieger anomaly
- O Peters Anomaly
- O Sclerocornea
- O Sturge Weber Syndrome
- O Neurofibromatosis type 1
- O Lowe Syndrome
- O Posterior anomalies
- O other

Signature of doctor (and readable name):
