# Supplementary material for: Childhood glaucoma registry in Germany: initial database, clinical care and research (pilot study)
Source: BMC Res Notes. 2022 Feb 10;15:32. doi: 10.1186/s13104-022-05921-8 (PMC8830121; doi:10.1186/s13104-022-05921-8)
Supplement: Supplementary file 4 — Additional file 4: Table S1. Causes and genetic data of secondary childhood glaucoma in Germany. [file 13104_2022_5921_MOESM4_ESM.docx]

**Additional file 4**

| **TABLE S1. Causes and genetic data of**  **secondary childhood glaucoma in Germany** | | | |
| --- | --- | --- | --- |
| **Diagnosis patients, n=17** | | | **revealed genes’ mutations** |
| Peters Anomaly  Aphakia | 7 (41%)  4 (24%) | | CYP1B1  CRYBB3; FYCO1 |
| Sclerocornea  Aniridia | 2 (12%)  1 (6%) | | TEK; GJA8  SOX11 |
| Axenfeld-Rieger anomaly  Sturge Weber Syndrome  Weill Marchesani Syndrome | 1 (6%) | | FOXC1 |
|  |  | 1 (6%)  1 (6%) | -  LTBP2 |
|  |  |  |  |
